# Conservation agricultural practices promoted arbuscular mycorrhizal fungal colonization and glomalin content on sandy clay loam of southern India

**DOI:** 10.1016/j.heliyon.2024.e41196

**Published:** 2024-12-17

**Authors:** Knight Nthebere, Tata Ram Prakash, Padmaja Bhimireddy, Latha P. Chandran, Jayasree Gudapati, Meena Admala, Kavuru Prasad

**Affiliations:** a Jayashankar Telangana State Agricultural University, *Hyderabad*, 500 030, *India*; b ICAR-IIRR, Indian Institute of Rice Research, *Hyderabad*, 500 030, *India*

**Keywords:** Arbuscular mycorrhizal fungi, Glomalin related soil protein, Soil quality, Conservation agriculture

## Abstract

Arbuscular mycorrhizal Fungi (AMF) are essential in agriculture and are often inter-linked with glomalin-related soil protein (GRSP) production which supports binding of aggregates, enhanced SOC and biological attributes. However, conservation agricultural practices in agroecosystem may have significant impact on AMF diversity, GRSP and soil quality-related parameters (SQRPs). This current experiment was implemented to gauge AMF conization percentage (AMF-CP), GSRP and significant changes on critical SQRPs, and to investigate the linkages between AMF-CP, GRSP and SQRPs as influenced by synergistic tillage and weed management in CA. Contrasting tillage practices (Main plots) included the **T**_**1**_:Conventional tillage with cotton- Conventional tillage with maize-fallow *i.e*., No *Sesbania rostrata* (Farmers’ practice), **T**_**2**_:Conventional tillage with cotton- Zero tillage with maize- Zero tillage with *Sesbania rostrata* and **T**_**3**_: Zero tillage with cotton + *Sesbania rostrata* residues- Zero tillage with maize + Cotton residues- Zero tillage with *Sesbania rostrata* + Maize stubbles. Weed management tactics (Sub plots) were **W**_**1**_: Chemical weed control, **W**_**2**_: Herbicide rotation, **W**_**3**_: Integrated weed management and **W**_**4**_: Single hand-weeded control. The roots and soil samples were collected at 60 DAS of maize. Analysis of examined parameters was done duly following standard protocols. The observed results indicated that 22.50 % of AMF-CP, 16.50 % of EE-GRSP and 12.08 % of T-GRSP was significantly higher in conservation tillage compared to **T**_**1**_ (Farmers practice). SQRPs followed similar trend in the case of tillage. Significantly higher SMBC and β-galactosidase (β-GaA) was exhibited by W_4_ and W_3_. A highly significant positive correlation of AMF-CP and T-GRSP pool with SOC (r = 0.951∗∗ and 0.756∗∗), SMBC (r = 0.872∗∗ and r = 0.761∗∗), β-GaA (r = 0.916∗∗ and r = 0.832∗∗) and WSA_1–2mm_ (r = 0.888∗∗ and 0.882∗∗), respectively was also observed. Generally, AMF-CP and GRSP, and SQRPs correlated positively and significantly. The following parameters; T-GRSP, SOC, WSA_1–2mm_, SMBC and β-GaA were selected as potential indicators to gauge the soil quality based on calculated principal component analysis (PCA). In general, the examined parameters in the study are highly supported by zero-till + crop residues, and can be considered indicators for monitoring agroecosystem.

## 1 Introduction

In recent times, a significant surge of population across the world inclusive of the semi-arid zones of southern India, and the subsequent high necessity for food compel the agriculturists to adopt industrial agricultural practices, thus threatening the sustainability of this farming practice [1]. This facilitates the soil degradation process. All these lead towards the decrement in soil's capacity to perform its ecological functions and ecosystem services, slow-down biological diversity and thus, increase environmental issues which ultimately result in poor soil quality and crop productivity. Therefore, there is an urgent need to encourage eco-friendly agricultural management practices to better make use of plant symbionts. For instance, arbuscular mycorrhizal fungi (AMF); soil quality indicator, has got various effects on ecosystem and can be used to assess ecosystem development brought about by imposed agricultural management practices [2]. Conservation agriculture (CA) has emerged as a promising revolutionary farming practice to be implemented not limited to semi-arid regions of India to gauge its effect on AMF and subsequent secretion of glomalin in soil, and other related soil quality parameters (soil microbial biomass carbon, β-galactosidase activity and soil organic carbon) which play a crucial part in soil aggregate stability. Conservation agriculture is defined as agricultural production strategy meant to bolster the sustainability of food production through preservation of soil and biological resources below and above the ground on a long-term basis with limited mechanical input [3], and it is based on these tenets; minimum soil disturbance, permanent soil cover through crop residues or cover crops, and crop rotations for achieving higher productivity. Minimal soil disturbances in zero tillage (ZT) practices with continual build-up of the crop residues on the soil surface all over the year may encourage AMF and abundance owing to enhanced establishment and preservation of extensive hyphal network. AMF is well-known to secrete considerable amount of glomalin content (GC) on hyphal network [4]. Glomalin is a constituent of soil organic matter (SOM) [[5], [6], [7]] that play a key role in the stabilization of the soil aggregates, soil organic carbon sequestration and improvement of the soil quality [8].

A number of research studies conducted in the previous four decades have apparently indicated that farmland management systems of cultivating non- AMF, diversified crops, protecting the crops and conventionally tilled soil, may influence AMF symbiosis, especially in cultivable soil [9]. In like manner, the content of glomalin may also differ among different natural environments, tillage methods [[10], [11], [12]] and on all parts of the crops colonized by one or distinct AMF species [13]. Intensive till has got a negative impact on soil structure which in turn may result in the shattering of AMF extraradical hyphal network, thus, leading to reduced glomalin concentration and other advantageous soil microbes [14,15], whereas no-till (NT) along-with crop residuals in conjunction with diversified crop species is thought to augment AMF biomass and SOC, thus resulting in better soil aggregation and increased glomalin content [4,16,17]. It is imperative to detect mycorrhizal percentage colonization within the roots of the crops and also quantify glomalin in soil as to ascertain the relationship between the two because the secretion of glomalin occurs within the roots by intra-radical hyphae [2,18]. Glomalin inter-connects with SOM and hence, it was more particularly called as “glomalin related soil protein (GRSP)” [2] and can be split-up into different proportions based on soil turn-over. The latest produced GC is computed as easily extractable-glomalin related soil protein (EE-GRSP) and another proportion is termed “total-GRSP (T-GRSP)” [19]. GC and β-Galactosidase activity (β-GaA) are other essential soil quality indicators (SQIs) [20,21]. β-GaA play a pivotal function in breaking down complex sugars and cycling of soil carbon [20,22]. Both GC and β-GaA could function more or less the same as β-GaA relates to GRSP and is directly influenced by AMF colonization [23].

Preservation of functional soil microbial biomass carbon (SMBC) is ascribed to β-GaA [4,22]. Apart from that, SMBC is also an important soil quality indicator (SQI) to identify early shifts in soil and stabilize carbon due to disturbance in soil caused by implemented agricultural management practices [24]. Thus, glomalin content is anticipated to correlate with SMBC and β-GaA. Since glomalin content (GC) is secreted by AMF hyphae, it is also anticipated that greater GC, SMBC and β-GaA could be impacted by farmland management practices with minimal soil disturbance. Limited studies conducted earlier have indicated the impact of distinct agricultural management practices on AMF, GC and associated soil quality parameters (SQPs) [4,25]. However, there are no reports available on extensive assessment of GC in soil, AMF colonization via microscopic observation and essentially related soil quality parameters as influenced by synergistic contrastive tillage practices and weed management options in conservation agricultural practices all over the world including semi-arid regions of southern India. Accordingly, the hypothesis of this present study is to investigate whether AMF, GRSP and associated soil quality parameters can function as logical indices for SQI under conservation agriculture. Thus, this current investigation was conducted to (1) assess the AMF conization, glomalin content secreted by AMF and significant changes on critical soil quality parameters and, (2) investigate the linkages between AMF colonization, GRSP and related important soil quality attributes as influenced by synergistic different tillage practices and weed management options in conservation agriculture at tasseling phase of the crop (maize) in cotton-maize-*Sesbania rostrata* cropping system.

## 2 Material and methods

### 2.1 Details of the experiment

This present on-going field experiment was executed at the college agricultural farm of PJTS Agricultural University in the Telangana Region of India located at 16^0^ 18′ 17″ North latitudes and 78^0^ 28′ 39″ East longitudes. The field experiment was implemented from 2020 in the monsoon with cotton (*Gossypium hirsutum*) followed by winter maize (*Zea mays*) in 2020–2021 and summer green manure (*Sesbania rostrata*) in 2021 in a sequence (rotation). Cotton is photo-thermo sensitive, maize is thermo sensitive, and *Sesbania rostrate* is legume cover crop. Hence, these crops were selected as the research objects in monsoon, winter, and summer seasons, respectively in accordance with the principles of conservation agriculture (CA) under semi-arid conditions of southern Telangana region. Weekly weather parameters (Supplementary Fig. 1) were recorded during maize advancement from the Agricultural Research Institute, Rajendranagar. The soil characterization with respect to distinct soil characteristics was achieved prior to the experiment *i.e*., before implementation and imposition of the treatments in 36 plots in 2020 through collection of 36 surface soil samples (0–15 cm soil depth) in triplicates (Table 1). These surface soil samples were all inter-mixed, processed and analyzed for parameters duly following the standard protocols and methods as shown in Table 1.

**Table 1:** Soil characterization in the 0–15 cm soil depth before the start of the experiment (2020), the methods and references used for each soil parameter analyzed.

S.No	Soil property	Soil test value	Method	Reference
1	Taxonomic categorization	*Inceptisol*	–	Soil Survey Staff [26]
2	**Mechanical separates**			
	Sand (%)	66.00	Hydrometer method	Bouyoucos [27]
	**SD**	0.30		
	**SE (m)±**	0.05		
	**CD (P=0.05)**	NS		
	Silt (%)	12.60		
	**SD**	0.06		
	**SE (m)±**	0.01		
	**CD (P=0.05)**	NS		
	Clay (%)	21.40		
	**SD**	0.42		
	**SE (m)±**	0.07		
	**CD (P=0.05)**	NS		
	Textural class	**Sandy clay loam**		
3	Slightly alkaline in soil reaction (pH)	7.82	Soil: water suspension (1: 2.5)	Jackson [28]
	**SD**	0.30		
	**SE (m)±**	0.05		
	**CD (P=0.05)**	NS		
4	Non- saline in electrical conductivity (dS m-₁)	0.33		
	**SD**	0.06		
	**SE (m)±**	0.01		
	**CD (P=0.05)**	NS		
6	Bulk density (Mg m^−3^)	1.23	Core sampler	Blake and Hartge [29]
	**SD**	0.24		
	**SE (m)±**	0.04		
	**CD (P=0.05)**	NS		
7	Medium in soil organic carbon (g kg^−1^)	6.50	Wet oxidation	Walkley and Black [30]
	**SD**	1.32		
	**SE (m)±**	0.22		
	**CD (P=0.05)**	NS		
8	Low in available soil nitrogen (kg ha-₁)	220.90	Alkaline KMnO_4_	Subbiah and Asija [31]
	**SD**	6.66		
	**SE (m)±**	1.11		
	**CD (P=0.05)**	NS		
9	Medium in available soil phosphorus (g kg-₁)	22.40	Olsen	Olsen et al. [32]
	**SD**	2.40		
	**SE (m)±**	0.40		
	**CD (P=0.05)**	NS		
10	High in available soil potassium (kg ha-₁)	408.75	1N Neutral ammonium acetate	Jackson [28]
	**SD**	3.60		
	**SE (m)±**	0.60		
	**CD (P=0.05)**	NS		

SD= Standard deviation, SE (m) **± = St**andard error of the mean, CD(P = 0.05) = Critical difference @ 5 % level of significance.

### 2.2 Experiment and treatment details

The study was carried-out in a split plot design in conservation agriculture. There were contrasting tillage systems (three) in main treatments (Table 2) and different weed control strategies (four) in sub treatments (Chemical weed control, Herbicide rotation, Integrated weed management (IWM) and Single hand-weeded control) as fully described in Supplementary Table 1. The treatment interactions of tillage and weed control were replicated thrice. The cumulative mean annual input of organic residues from cotton and *Sesbania rosrata* retained in conservation tillage plots (T_3_), since the year 2020–2023, was about 200.0–240.0 Mg ha^−₁,^ estimated according to Bolinder et al. [33].

**Table 2:** Tillage treatment details with crop diversification in the main plots.

Tillage (s)	Seasons		
	Monsoon	Winter	Summer
T_1_:	CT (C) -	CT (M) -	Fallow (N*Sr*)
T_2_:	CT (C) -	ZT (M) -	ZT (*Sr*)
T_3_:	ZT(C) + *Sr*R -	ZT (M) + CR -	ZT (*Sr*) + MS

CT(C) = conventional tillage (cotton), ZT(M) = zero tillage (maize), ZT(C) + *Sr*R = zero tillage (cotton) + *Sesbania rostrate* residues, Fallow (N*Sr*) = Fallow(No *Sesbania rostrata*), ZT(*Sr*) = zero tillage (*Sesbania rostrata*), ZT(C) + *Sr* = zero tillage (cotton) + *Sesbania rostrata* residues, ZT (M) + CR = zero tillag (Maize) + cotton residues, ZT (*Sr*) + MS = zero tillage (*Sesbania rostrata*) + maize stubbles.

## 3 Crop management practices

The experimental particulars and attributes of crop varieties are shown in Supplementary Tables 2 and 3, respectively. Before sowing of the crops (cotton and maize), the field was plowed twice followed by rotovation and levelling field operators in conventionally tilled (T_1_) plots, whereas in no-till (ZT) plots, seeds dibbling was performed. For *Sesbania*, sowing was done directly in solid rows (30 cm spacing) between the maize stubbles in the T_2_ and T_3_ treatments without any tillage operations. Conversely, the CT (T_1_) plots were fallowed during summer *i.e*., there was no *Sesbania* in such plots. This distinction in management practices reflects the specific treatments applied to each plot in the experimental design. The crops particularly cotton and maize were raised in accordance with recommended dose of fertilizers (RDFs), cultural practices and typically advanced with rainfall in monsoon and supplemental irrigation in winter. At 30 days after sowing (DAS), *Sesbania rostrata* was knock-down and removed in the T_2_ while in the T_3_, shrub master was used to shred and retain *Sesbania* as surface mulch to the soil. The details on the dates of sowing and harvesting for each crop are presented in Supplementary Table 4.

## 4 Sampling

### 4.1 Rhizosphere soil and roots

The rhizosphere soil was collected from the root zone and root samples from the plant at distinct spots in 36 plots each by pulling out the plant, separating the roots from the plant through cutting with the help of a knife followed by removing rhizosphere soil then kept in a polythene zip cover [34] during 2023 at tasseling stage of maize (8th cropping cycle). The rhizosphere soil samples were subsequently inter-mixed, unified, and composed into composite sample (s) per plot. Similarly, the roots were garnered from three maize plants per plot, washed gently under tap running water; drained excess moisture adhered to the roots. These roots were stored at 4 ^⸰^C to be processed for AMF/root colonization [4]. The whole composite rhizosphere soils from each treatment were sub-divided into 2, kept in polythene bags with zippers in the laboratory, of which one portion was stored at 4 ^⸰^C to be utilized for β-GaA and SMBC analysis, and the remaining portion was well air-dried under shade, passed through 2 mm and 0.5 mm sieves to be utilized for glomalin related soil protein (GRSP) and soil organic carbon (SOC), respectively. For soil aggregate stability, clods were collected from respective plots and stored in the laboratory for analysis.

## 5 Soil and root standard analytical procedures

### 5.1 Assessment and computation of AMF colonization percentage

To stain and assess AMF colonization, 2 g of fresh fine lateral roots were utilized. Root samples were cleared and stained according to Phillips and Hayman [35]. About 10 segments were arranged in a microscopic slide 10 times and examined for the presence of hyphae, arbuscules and vesicles under optical microscope at 20 megapixels following the grid line-intersect method [36] (Giovannetti and Mosse, 1998). In general, 100 segments of the roots were considered to estimate percentage root colonization (% AMF colonization) according to equation (1).

(1) $$\text{Percentage}\;\left(\%\right)\;\text{AMF}\;\text{colonization}=\frac{Number\;of\;root\;bits\;with\;injection}{Number\;of\;root\;bits\;examined}\times 100$$

### 5.2 Extraction and computation of glomalin

The two pools of glomalin *i.e*., easily extractable-glomalin soil related protein (EE-GRSP) and total-GRSP (T-GRSP) were determined according to Wright and Updahyaya [37]. The quantity and/or absorbance of glomalin within the supernatant was determined spectrophotometrically (λ = 595 nm) by the Bradford dye-binding test [37] and expressed as mg g^−1^ of soil.

### 5.3 Soil organic carbon (SOC), microbial biomass carbon (SMBC) and β-galactosidase activity

The analysis for SOC was achieved by wet oxidation method [30] (Walkley and Black, 1934). SMBC was determined according to fumigation extraction method [38,39]. The β-galactosidase (β-GaA) was estimated according to Eivazi and Tabatabai [40] and determined by spectrophotometry (λ = 420 nm). The content of moisture in soil was estimated duly following the protocol given by Wu et al. [41], and the information was employed to quantify SMBC and β-GaA.

### 5.4 Soil aggregate stability

The macro-aggregates of 1.0–2.0 mm were deemed for examination because of sensitivity towards short-term agricultural management practices and also lesser number of years of soil treatment [42]. To achieve this analysis, soils were sieved prior as to retain the soil fragments of not more than 2 mm and, were kept air-dried until further analysis. 5.0 g of soil aggregates (clods) in replicates per plot were moistened by capillary action for 15 min and water stable aggregates were analyzed by wet-sieving method [42].

## 6 System yield in terms of cotton equivalent yield

Grain yield for maize in each net plot was recorded by weighing air-dried produce at 14 % moisture content before threshing and expressed in kg ha^−1^. The system yield was computed in terms of cotton equivalent yield considering monsoon seed cotton and grain/kernel yield after 3rd-year of the experiment (Supplementary Table 5) as under (Equation (2)):

(2) $$\text{System}\;\text{CEY}\;\left(\text{kg}\;{\text{ha}}^{-1}\right)=\text{Economical}\;\text{yield}\;\text{of}\;a\;\text{maize}\;\text{crop}\;\left(\text{kg}\;{\text{ha}}^{-1}\right)\;x\;\frac{Price\;\left(\text{RS}\;{\text{Kg}}^{-1}\right)\;of\;same\;crop\;i.e.,maize}{\mathrm{Pr}\;ice\;\left(\text{RS}\;{\text{Kg}}^{-1}\right)\;of\;cotton}$$

## 7 Statistical analysis

The two-way ANOVA for split-plot experiments was used to discriminate and examine critical difference among the treatment means for their significance at 5 % probability [43]. Duncan multiple rank test (DMRT) was also performed to rank the treatment means at 5 % probability level. Pearson's correlation coefficients and principal component analysis (PCA) were performed in SQI CAL software (a tool for soil health assessment) developed by Mohanty [44] to find the relationship between system cotton equivalent yield, AMF colonization percentage, glomalin related soil protein (GRSP) and some soil quality related parameters (SOC, SMBC, β-Galactosidase activity and water stable aggregates size of 1–2 mm), and to identify the selected variables from respective PCs that could be considered for assessing the soil quality as influenced by conservation agricultural management practices (tillage and weed management).

## 8 Results

### 8.1 Arbuscular mycorrhizal fungi (AMF) and glomalin related soil protein (GSRP)

The perusal of data on AMF has clearly demonstrated that adopting distinct tillage and weed management options in conservation agriculture management practice during maize (8th cropping cycle) in cotton-maize-*Sesbania rostrata* cropping system, promoted AMF (Supplementary Fig. 2). Among tillage practices, AMF colonization percentage was 22.50 % and 12.50 % significantly higher in the ZT(C) + *Sr*R-ZT(M) + CR-ZT(*Sr*) + MS (Conservation tillage) system over CT(C)-CT(M)-Fallow(N*Sr*) *i.e*., Farmers practice and CT(C)-ZT(M)-ZT(*Sr*) systems, respectively (Table 3). Across all the tillage practices examined, the easily extractable-GRSP (EE-GRSP) and total- GRSP (T-GRSP) fractions followed trends similar to that of AMF colonization percentage (Table 3).

**Table 3:** Impact of tillage practices and weed management options in conservation agriculture on colonization of AMF (AMF-C), easily extractable- (EE-GRSP) and total-glomalin related soil protein (T-GRSP), organic carbon (OC), and water stable aggregates of 1.0–2.0 mm (WSA_1.0–2.0 mm_) after 3rd year in the 8th cropping (maize) cycle.

Treatment Interaction		AMF-C (%)	EE-GRSP (mg g-₁)		T-GRSP (mg g-₁)		OC (g kg^−1^)		WSA_1–2mm_ (%)	
Tillage	WM									
T_1_: CT(C)-CT(M)-Fallow (N*Sr*)	W_1_	45.00	0.89		2.72		6.50		47.40	
	W_2_	45.00	0.71		2.56		6.53		47.60	
	W_3_	50.00	0.69		2.54		6.88		48.96	
	W_4_	50.00	0.94		2.67		6.93		50.00	
	W_1_	55.00	0.93		2.78		7.15		57.45	
T_2_: CT(C)-ZT(M)-ZT(*Sr*)	W_2_	60.00	0.85		2.88		7.17		57.65	
	W_3_	60.00	1.06		2.90		7.25		58.90	
	W_4_	55.00	0.92		3.08		7.28		60.25	
T_3_: ZT(C) + *Sr*R-ZT(M) + CR-ZT(*Sr*) + MS	W_1_	65.00	0.94		2.93		7.56		64.55	
	W_2_	65.00	0.92		3.01		7.80		64.88	
	W_3_	65.00	1.00		3.03		7.97		65.98	
	W_4_	85.00	1.02		2.95		8.35		67.52	
**Tillage practices**										
T_1_: CT(C)-CT(M)-Fallow (N*Sr*)	47.50		0.81		2.62		6.71		48.49	
T_2_: CT(C)-ZT(M)-ZT(*Sr*)	57.50		0.94		2.91		7.21		58.56	
T_3_: ZT(C) + *Sr*R-ZT(M) + CR-ZT(*Sr*) + MS	70.00		0.97		2.98		7.92		65.73	
**Weed Management options**										
W_1_- Chemical weed control	55.00		0.92		2.81		7.07		56.47	
W_2_- Herbicide rotation	56.67		0.83		2.82		7.17		56.71	
W_3_- IWM	58.33		0.92		2.82		7.37		57.95	
W_4_- Single hand-weeded control	63.33		0.96		2.90		7.52		59.26	
	**SE(m)±**	**CD(P=0.05)**	**SE(m)±**	**CD(P=0.05)**	**SE(m)±**	**CD(P=0.05)**	**SE(m)±**	**CD(P=0.05)**	**SE(m)±**	**CD(P=0.05)**

**Tillage**	1.667	6.719	0.008	0.032	0.012	0.050	0.150	0.570	1.194	4.689
**Weed Management**	2.485	NS	0.033	NS	0.053	NS	0.190	NS	1.106	NS
**Interactions**										
**W at same level of T**	3.333	NS	0.016	NS	0.025	NS	0.24	NS	1.915	NS
**T at same level of W**	4.082	NS	0.050	NS	0.081	NS	0.25	NS	2.044	NS

T_1_ = conventional tillage (cotton) – conventional tillage (maize) – Fallow (No *Sesbania rostrata*), T_2_ = conventional tillage (cotton) – zero tillage (maize) – zero tillage (*Sesbania rostrata*), T_3_ = zero tillage (cotton) + *Sesbania rostrata* residues (*Sr*R)– zero tillage (maize) + cotton residues (CR) – zero tillage (*Sesbania rostrata*) + maize stubbles (MS), T = TillIage, W=Weed management, IWM=Integrated weed management, CD (P = 0.05) = critical difference at 5 % probability level, NS = non-significant, SE(m) = standard error of the mean.

The rate of decrease on EE-GRSP was 3.09 % and 16.50 % in the CT(C)-ZT(M)-ZT(*Sr*) and Farmers practice, respectively compared to Conservation tillage. In like manner, the T-GRSP contents decreased by 2.35 % and 12.08 % under CT(C)-ZT(M)-ZT(*Sr*) and Farmers practice, respectively relative to Conservation tillage practice. The significant percentage contribution of EE-GRSP pool to T-GRSP was 30.92 %, 32.30 %, and 32.55 % in the Farmers practice, CT(C)-ZT(M)-ZT(*Sr*) and Conservation tillage system, indicating higher turn-over level of EE-GRSP under Conservation tillage followed by CT(C)-ZT(M)-ZT(*Sr*) compared to Farmers practice. The observations on AMF colonization percentage, EE-GRSP and T-GRSP as influenced by weed management options and the treatment interactions (tillage practices-weed management options) showed no significant difference (Table 3).

### 8.2 Soil organic carbon, microbial biomass carbon and β-Galactosidase activity

**Fig. 1:**
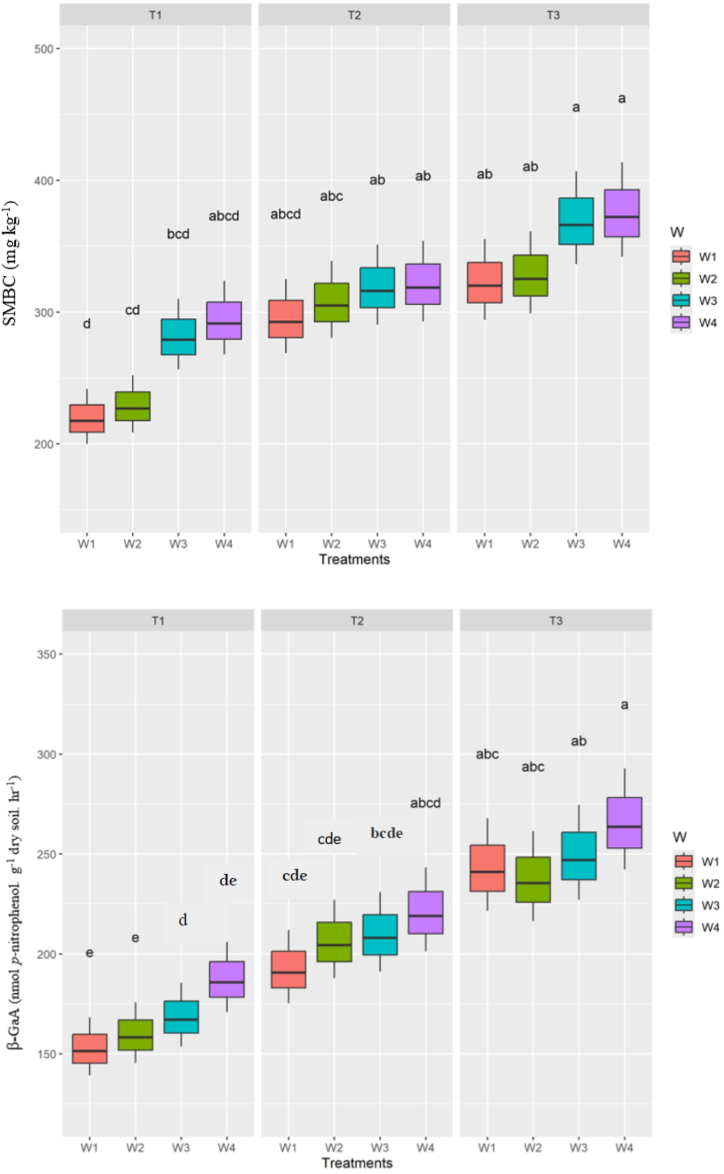
Impact of tillage practices and weed management options in conservation agriculture (CA) on soil microbial biomass carbon **(SMBC)** and β-galactosidase activity **(β-GaA)**. Means having distinct letters demonstrate significant variances between the treatments at 5 % probability level (Duncan multiple rank test) and means having the same letters indicate no significant variances among the treatment means at 5 % probability level. Refer to Supplementary Table 1 and Table 2 for treatment details.

The content of soil organic carbon (SOC) in the 0–15 cm significantly increased by 8.97 % and 15.28 % under the ZT(C) + *Sr*R-ZT(M) + CR-ZT(*Sr*) + MS (Conservation tillage) practice in comparison with the CT(C)-ZT(M)-ZT(*Sr*) and CT(C)-CT(M)-Fallow(N*Sr*) *i.e*., Farmers practice, respectively. No significant effect by weed management and treatment interactions (tillage-weed management) was observed on SOC (Table 3). The soil microbial biomass carbon (SMBC) and β-Galactosidase activity (β-GaA) were significantly enhanced by adoption of Conservation tillage compared to other tillage practices examined (Fig. 1). Among weed management options, a significant increase on SMBC and β-GaA was observed with single hand-weeded control, followed by integrated weed management (IWM) relative to chemical weed control and herbicide rotation sub-treatments (Fig. 1). Tillage and weed management interaction effects on SMBC and β-GaA were significantly higher with adoption of the conservation tillage in combination with Singe hand-weeded control followed by Conservation tillage on interaction with IWM compared to all other treatment combinations examined (Fig. 1).

### 8.3 Water stable aggregate

The WSA_1–2mm_ percentage was significantly greater in the Conservation tillage relative to other tillage practices under the study (Table 3). The rate at which WSA_1–2mm_ decreased in the CT(C)-ZT(M)-ZT(*Sr*) and Farmers practices was 10.91 % and 26.23 %, respectively relative to the Conservation tillage practice. Weed management options and treatment interactions (tillage-weed management) effects on WSA_1–2mm_ were non-significant (Table 3).

## 9 System cotton equivalent yield

The maize grain yield obtained from different tillage-weed management combinations, was converted into cotton equivalent yield (CEY) considering the monitory equivalence. The winter cotton equivalent yield was subsequently added to the monsoon cotton yield of the 3rd year to arrive at the CEY of the cotton– maize system *i.e*., system yield after 3 years. The ZT(C) + *Sr*R-ZT(M) + CR-ZT(*Sr*) + MS exhibited a significantly higher CEY (3775 kg ha^−1^) relative to other tillage practices examined (Table 4). Among the weed management strategies, integrated weed management (IWM) had a significantly greater system CEY (4157 kg ha^−1^) compared to the rest of the weed management practices examined. Based on the tillage and weed management combinations, the ZT(C) + *Sr*R-ZT(M) + CR-ZT(*Sr*) + MS with IWM, recorded significantly higher CEY (4453 kg ha^−1^), while the combination of CT(C)-ZT(M)-ZT(Sr), CT(C)-CT(M)-Fallow(N*Sr*) and ZT(C) + *Sr*R-ZT(M) + CR-ZT(*Sr*) + MS with Single hand-weeded control resulted in the lowest CEY values (1848 kg ha^−1^, 1767 kg ha^−1^ and 2157, respectively).

**Table 4:** System yield in terms of system cotton equivalent yield (CEY) as influenced by tillage and weed management (WM) practices after 3rd year (8th crop cycle).

Treatment Interaction	WM	System (CEY) (kg ha^−₁^)
Tillage		
T_1_: CT(C)-CT(M)-Fallow (N*Sr*)	W_1_	3756
	W_2_	3801
	W_3_	3908
	W_4_	1848
	W_1_	4005
T_2_: CT(C)-ZT(M)-ZT(*Sr*)	W_2_	4187
	W_3_	4109
	W_4_	1767
T_3_: ZT(C) + *Sr*R-ZT(M) + CR-ZT(*Sr*) + MS	W_1_	4292
	W_2_	4206
	W_3_	4453
	W_4_	2157
**Tillage practices**		
T_1_: CT(C)-CT(M)-Fallow (N*Sr*)	3328	
T_2_: CT(C)-ZT(M)-ZT(*Sr*)	3517	
T_3_: ZT(C) + *Sr*R-ZT(M) + CR-ZT(*Sr*) + MS	3775	
**Weed Management options**		
W_1_- Chemical weed control	4018	
W_2_- Herbicide rotation	4065	
W_3_- IWM	4157	
W_4_- Single hand-weeded control	1921	
	**SE(m)±**	**CD(P=0.05)**

**Tillage**	18.69	73.38
**Weed Management**	40.29	119.71
**Interactions**		
**W at same level of T**	69.79	207.35
**T at same level of W**	63.26	187.96

T_1_ = conventional tillage (cotton) – conventional tillage (maize) – Fallow (No *Sesbania rostrata*), T_2_ = conventional tillage (cotton) – zero tillage (maize) – zero tillage (*Sesbania rostrata*), T_3_ = zero tillage (cotton) + *Sesbania rostrata* residues (*Sr*R)– zero tillage (maize) + cotton residues (CR) – zero tillage (*Sesbania rostrata*) + maize stubbles (MS), T = TillIage, W=Weed management, IWM=Integrated weed management, CD (P = 0.05) = critical difference at 5 % probability level, NS = non-significant, SE(m) = standard error of the mean.

## 10 Relationship between AMF percentage colonization, glomalin with other soil quality related parameters and system cotton equivalent yield

The Pearson correlation coefficient analysis was performed to ascertain the associateship of the crop yield in terms of system yield and conservation agricultural management practices with arbuscular mycorrhizal fungi colonization percentage (AMF-CP), glomalin related soil protein (GRSP) and other soil quality related parameters (soil organic carbon, soil microbial biomass carbon, β-Galactosidase activity and water stable aggregates of 1–2 mm). The associateship among the AMF-CP, GRSP (EE-GRSP and T-GRSP), and SOC, SMBC, β-galactosidase activity and water stable aggregates of 1–2 mm (WSA_1–2mm_) was significantly positive (Table 5). There was a significant positive correlation observed between AMF-CP with EE-GRSP and T-GRSP (r = 0.611∗ and r = 0.662∗, respectively). In like manner, correlation between EE-GRSP and T-GRSP was positive and highly significant (r = 0.719∗∗) (Table 5). The AMF-CP and T-GRSP correlation with all associated soil quality parameters examined (SOC, SMBC, β-GaA and WSA_1–2mm_) was significantly higher compared to the EE-GRSP thereof. No significant correlation was observed between system yield and all the parameters examined in the present study (Table 5).

**Table 5:** Correlation matrix of AMF-CP, EE-GRSP, T-GRSP and other essential related soil quality indicators (SOC, SMBC, β-GaA and WSA_1–2mm_) as influenced by contrasting tillage practices and weed management options in conservation agriculture (CA) after 3rd year during maize (in the 8th cropping cycle).

	AMF-CP	EE-GRSP	T-GRSP	SOC	β-GaA	SMBC	WSA_1–2mm_	SY CEY
AMF-CP	1							
EE-GRSP	0.611^a^	1						
T-GRSP	0.662^a^	0.719^b^	1					
SOC	0.951^b^	0.633^a^	0.756^b^	1				
β-GaA	0.916^b^	0.664^a^	0.832^b^	0.958^b^	1			
SMBC	0.872^b^	0.657^a^	0.761^b^	0.947^b^	0.929^b^	1		
WSA_1–2mm_	0.888^b^	0.677^a^	0.882^b^	0.948^b^	0.974^b^	0.909^b^	1	
SY CEY	−0.084^NS^	−0.156^NS^	−0.057^NS^	−0.059^NS^	−0.079^NS^	−0.121^NS^	0.059^NS^	1

AMF-CP = arbuscular mycorrhizal fungi colonization percentage, EE-GRSP = easily extractable glomalin related soil protein, T-GRSP = total glomalin related soil protein, SOC = soil organic carbon, SMBC = soil microbial biomass carbon, β-GaA = β-galactosidase activity and WSA_1–2mm_ = water stable aggregates of 1–2 mm, SY CEY = system yield in terms of cotton equivalent yield.

a correlation is significant (p < 0.01).

b correlation is highly significant (p < 0.05).

## 11 Principal component analysis (PCA) and variable selection

PCA was employed to analyse arbuscular mycorrhizal fungi colonization percentage, easily extractable and total glomalin related soil protein, soil organic carbon, soil microbial biomass carbon, β-galactosidase activity and water stable aggregates of 1–2 mm as to ascertain their contribution for improving soil quality. The PCA clustered all the observations into 6 principal components (PCs) (Table 6). PC 1 contributed 85.16 % variance with Eigen value of 5.11 and was the only PC with Eigen value of more than 1 in comparison with the other PCs (Table 6). Therefore, PC 1 was regarded as the best PC for further analysis. The weight values for respective PCs was 1 because both variable and cumulative variable percent was 85.16 % for PC 1. As numerous data sets are dependent, the indicators or variables were selected based on PCA and correlation among the soil parameters. These selected variables from PC 1 were total glomalin related soil protein, soil organic carbon, β-galactosidase activity, soil microbial biomass carbon and water stable aggregates of 1–2 mm based on their higher factor loading scores (Table 6).

**Table 6:** Principal component analysis and selection of potential variables for improving soil quality.

PC			Variable selection ( ± of 10 % Max)				
	Eigenvalue	% variance	Column	Variable	Column_For_Scoring		Factor loadings
1	5.11	85.16	1	T-GRSP	2		0.89
			1	SOC	3		0.95
			1	β-GaA	4		0.97
			1	SMBC	5		0.95
			1	WSA_1–2mm_	6		0.98
2	0.51	8.48					
3	0.26	4.28					
4	0.08	1.27					
5	0.03	0.51					
6	0.02	0.28					

PC = principal component, T-GRSP = total glomalin related soil protein, SOC = soil organic carbon, SMBC = soil microbial biomass carbon, β-GaA = β-galactosidase activity and WSA_1–2mm_ = water stable aggregates of 1–2 mm.

## 12 Discussions

### 12.1 AMF, glomalin and soil organic carbon as influenced by tillage and weed management practices in conservation agriculture

Adoption of conservation agriculture has clearly demonstrated the capability of conservation tillage system (zero tillage + retention of crop residues) to bolster inhabitant AMF and other soil quality related parameters (SOC, SMBC, β-galactosidase activity and water stable aggregate size of 1–2 mm). A minimally disturbed soil environment alike zero tillage with well-managed crop residues as examined in this present investigation has apparently exhibited a significantly higher AMF colonization percentage (AMF-CP) over conventionally tilled soils. This greater AMF-CP is ascribed to the intact filamentous fungal network and a shift in the AMF structural populace under no-till which bolsters greater hyphal volume. Besides, Glomus species being the game changer to prepotent maize roots colonization [4] could probably contribute towards enhancing AMF-CP in conjunction with conservation tillage (ZT(C) + *Sr*R- ZT(M) + CR-ZT(*Sr*) + MS). The contents of glomalin followed the trend alike AMF-CP being significantly greater under conservation tillage. The decline in glomalin content (GC) observed in the CT(C)-CT(M)-Fallow (N*Sr*) (farmers practice) and CT(C)-ZT(M)-ZT(*Sr*) concur with the findings of Bedini et al. [45] who have reported the decline in GC under continuously tilled monoculture maize experimental plots although the field trial was done in the temporal zones. Earlier studies have apparently shown that tilling of the soil creates disturbance which ultimately result in decreased root colonization [46] accompanied by a greater interference in the extra-radical mycelium. This could directly lead towards the declined glomalin content in soil.

The AMF-CP and SOC followed the same trend and were significantly greater under conservation tillage system. Adoption of zero tillage coupled with crop residues retention promote accumulation of biomass in the soil. Through rhizodeposition, approximately forty percent of photosynthetically stable carbon goes into the soil environment [47] which can considerably contribute towards carbon. Other than that, the carbon held in soil carbohydrates considerably contributes up to 20 % of soil organic matter (SOM) [47]. In like manner, glomalin-carbon having glycoprotein is anticipated to considerably supply SOM. This suggests greater SOC and GRSP in the treatment(s) with higher AMF-CP. This is inconsistent with previous findings in which the greater fractions of GRSP were found in the zero tillage [15].

### 12.2 Water stable aggregates, soil microbial biomass carbon and β-galactosidase activity (β-GaA) as influenced by tillage and weed management in conservation agriculture

It is evident that other relevant soil quality related parameters (SQRPs) *i.e*., soil microbial biomass carbon (SMBC), β-galactosidase activity **(**β-GaA) and water stable aggregate size of 1–2 mm (WSA_1–2mm_) examined in the present study were also found to be significantly greater under conservation tillage plots. While weed management in conservation agriculture is of utmost importance, it is recommended to manage the weeds eco-friendly as to minimise the negative impact of applying herbicides to the soil environment on critical and sensitive soil biological properties. The SMBC and β-GaA were significantly enhanced by single hand-weeded control followed by integrated weed management (IWM) relative to herbicides treated plots (chemical weed control and herbicide rotation) (Table 3). This might be ascribed to conducive biophysical environment created for preponderance of rhizosphere soil microorganisms. Similarly, Manaswini et al. [48] and Nthebere et al. [49] announced the same results in which manual weeding at once and manual weeding in combination with the application of post-emergence herbicides (integrated weed management) in corn field resulted in an increased microbial activity.

The improvement in these parameters examined thereof was observed under these plots could be associated with carbohydrates liberated by glomalin soil related protein (GSRP) which functions as a trap for diverse group of microorganisms and SOM, and finally resulting in the increased SMBC, β-GaA and development of extra-cellular polycarbohydrates to stabilizes water aggregates size of 1–2 mm [24,50]. The significant reduction observed in WSA_1–2mm_ under CT(C)-CT(M)-Fallow (N*Sr*) and CT(C)-ZT(M)-ZT(*Sr*) (Table 3) concur with the findings of Beare et al. [51] who have indicated that continuous cultivation of the soil leads to decreased soil aggregation. Wright et al. [52] have also reported better stability of 1–2 mm size aggregates after three years of no-till (NT) relative to plough-tillage. However, biological properties bind the soil particles and quickly respond to soil disturbances and other agricultural management practices. Of all the biological properties examined, AMF is of critical importance, ascribed to a physical impact of a network enclose to the soil fragments, along-with the hyphal secretion of some GRSP, which binds the soil constituent [6,46].

### 12.3 System cotton equivalent yield

The system yield in terms of cotton equivalent increased by nearly 14 % with zero tillage + residue retention treatment and by 116 % with weed management interventions. It was also observed that system yield was higher with integrated weed management (IWM), followed by the herbicide application. This could be due to the herbicides ability for weed suppression, which reduces weed competition, thus giving access to the plant for adequate resources (moisture, air, light and nutrients) required for the growth and development. On the other hand, herbicides decrease soil biological attributes, particularly in regions likes semi-arid of southern Telangana in India with inadequate status of SOC and alkaline soil pH probably due to inhibition of soil C cycling. Similar results were reported by Rose et al. [53] who has observed inhibition of soil biota in alkaline and low organic matter soils treated with glyphosate and atrazine in the semi-arid zones of Australia. So, considering both system yield and soil quality related attributes examined in the present study, IWM along-with ZT(C) + *Sr*R-ZT(M) + CR-ZT(*Sr*) + MS was considered remunerative treatment combination with better system yield, soil microbial biomass carbon and β-galactosidase activity probably brought about by low weed density at the initial crop developmental phase. The application of herbicides in time and subsequent control of later germinated weeds by the supplemented inter-cultural operation followed by hand weeding cause a reduction in weed competition, leading to increased photosynthetic activity [54,55], and this agrees with the results of the present investigation.

### 12.4 Correlation between AMF percentage colonization, glomalin with other soil quality related parameters and system cotton equivalent yield

All contrasting tillage and weed management practices examined in current investigation, apparently demonstrated a positive correlation of GRSP with AMF-CP probably due to larger segment (approximately 80 %) of glomalin which is available on the spores and filaments [56] elucidating the positive correlation of GRSP with AMF-CP. The highly positive and significant correlation which existed between EE-GRSP and T-GRSP fractions in this present investigation aligns with the previous studies [57]. This is likely the result of EE-GRSP fraction being a component of recalcitrant GRSP and thus, a highly positive correlation is anticipated among the two [46]. A potent and positive correlation which was observed to exist between AMF-CP, SOC and other soil properties involved in carbon cycling in this current study, has also been reported in the previous studies [19,58]. Further, the experimental findings have identified GRSP as a crucial constituent of SOC [59,60]. System cotton equivalent yield did not significantly correlate with any of the soil parameters examined in the present study. This could be due to crop yield determinant factors such as moisture, sunlight etc. These results concur with Schneider et al. [61] who observed no significant correlation between soil biological related parameters with corn-soybean yields in a cultivated soil. Non-significant correlation between tomato yields and biological parameters was also reported by Yankit et al. [62] in the mid-hill zone of Himachal Pradesh of India attributed to variability in tomato yield over the 2-years experiment.

### 12.5 Principal component analysis and variable selection

The role of SOC is known to alter and bolster many soil functions such as enzyme activities, glomalin, SMBC and soil aggregation etc. Fitly, SOC has been associated and correlated positively with enzyme activities, glomalin, mean weight diameter and SMBC among other soil parameters at 0.05–0.01 significance levels [[63], [64], [65]]. This could be ascribed to improved soil health owing to adoption of conservation agriculture practice. The observed selected variables in PC 1 (Table 6) suggest that soil quality can better be gauged through assessment of total glomalin related soil protein, SOC, SMBC, water aggregates size of 1–2 mm and β-galactosidase activity in conservation agricultural system under cotton-maize-*Sesbania rostrata* system in the semi-arid regions of Southern India.

## 13 Conclusion

Conservation tillage (ZT(C) + *Sr*R-ZT(M) + CR-ZT(*Sr*) + MS) agricultural management practice, adopted for three consecutive years in the 5th cropping cycle (maize) has significantly resulted in an increased arbuscular mycorrhizal colonization percentage (AMF-CP), turn-over rates of glomalin, other associated soil quality parameters *viz*., soil organic carbon (SOC), soil microbial biomass carbon (SMBC), β-galactosidase activity (β-GaA) and water stable aggregate size of 1–2 mm (WSA_1–2mm_). This is confirmed by a significant positive correlation among AMF-CP, glomalin content, OC, SMBC, β-GaA and water stable aggregate size of 1–2 mm. The biological factors (SMBC and β-GaA) were significantly enhanced by single hand-weeded control followed by integrated weed management in case of weed control practices. Based on the results of principal component analysis (PCA) obtained in the present study, it may be deduced that the total glomalin soil related protein, SOC, WSA_1–2mm_, SMBC and β-GaA could be very essential criterion to assess soil quality and the development of agro-ecosystem under implemented CA management tactics in this zone. Further research is necessitated to gauge the soil quality index and the determinants of AMF biomass in addition to AMF diversity and GRSP which would aid towards developing a new tactic of maintaining the soil quality under conservation agricultural management practice in the semi-arid Indian zone.

## CRediT authorship contribution statement

**Knight Nthebere:** Methodology, Investigation, Formal analysis, Data curation, Conceptualization. **Tata Ram Prakash:** Methodology, Investigation, Formal analysis, Data curation, Conceptualization. **Padmaja Bhimireddy:** Supervision, Methodology, Investigation, Formal analysis, Data curation, Conceptualization. **Latha P. Chandran:** Methodology, Investigation, Formal analysis, Data curation, Conceptualization. **Jayasree Gudapati:** Writing – review & editing, Writing – original draft, Visualization, Validation. **Meena Admala:** Validation, Formal analysis, Data curation. **Kavuru Prasad:** Writing – original draft, Validation, Resources.

## Ethics approval

Review and/or approval by an ethics committee was not needed for this study because there was no human and animal participation involved.

## Competing interests

The authors declared no competing interests.

## Data Availability Statement

The datasets generated and analyzed during the current study are not publicly available, but are available from the corresponding author on reasonable request.

## Funding statement

Author(s) declare that no funding was received.

## Declaration of competing interest

The authors declare the following financial interests/personal relationships which may be considered as potential competing interests: Knight Nthebere reports financial support was provided by All India Coordinated Research Project on Weed Management. Knight Nthebere reports a relationship with Professor Jayashankar Telangana State 10.13039/100020623Agricultural University that includes: funding grants and non-financial support. Tata Ram Prakash reports a relationship with Professor Jayashankar Telangana State 10.13039/100020623Agricultural University that includes: employment and funding grants. Gudapati Jayasree reports a relationship with Professor Jayashankar Telangana State Agricultural University that includes: employment. Bhimireddy Padmaja reports a relationship with Professor Jayashankar Telangana State Agricultural University that includes: employment. Meena Admala reports a relationship with Professor Jayashankar Telangana State Agricultural University that includes: employment. Latha P Chandran reports a relationship with Indian Institute of Rice Research that includes: non-financial support. If there are other authors, they declare that they have no known competing financial interests or personal relationships that could have appeared to influence the work reported in this paper.
