# Supplementary material for: Conservation agricultural practices promoted arbuscular mycorrhizal fungal colonization and glomalin content on sandy clay loam of southern India
Source: Heliyon. 2024 Dec 17;11(1):e41196. doi: 10.1016/j.heliyon.2024.e41196 (PMC11728879; doi:10.1016/j.heliyon.2024.e41196)
Supplement: Multimedia component 1 [file mmc1.doc]

**Supplementary Table 1.** Weed management (W) in sub-treatments and interaction with tillage (T) in main treatments

|  | **Monsoon (Cotton)** | | | | **Winter (Maize)** | | | |
| --- | --- | --- | --- | --- | --- | --- | --- | --- |
|  | **W_1_:**  **Chemical Weed Control** | **W_2_: Herbicide Rotation (Alternative year)** | **W_3_:**  **IWM** | **W_4_:**  **Single hand- weeded Control** | **W_1_:**  **Chemical Weed Control** | **W_2_: Herbicide Rotation**  **(Alternative year)** | **W_3_: IWM** | **W_4_:**  **Single hand- weeded Control** |
| **T_1_** | Diuron  pre-emergence (PE) application 0.75 kg/ha *fb* tank mix appli-cation of pyrithiobac  -sodium 62.5 g/ha+ quiza- lofop-ethyl 50 g/ha as PoE (Post-emergen- ce application) (2-3 weed leaf stage) *fb* directed spray (inter-row) of paraquat 0.5  kg/ha at 50-55 DAS. | Diuron PE 0.75  kg/ha *fb* tank mix application of pyrithiobac-sodium  62.5 g/ha+quizalofop-ethyl 50 g/ha as PoE (2-3 weed leaf stage) *fb* directed spray (inter-row) of paraquat 0.5 kg/ha at 50-55 DAS.  **rotated with** Pendimethalin 1 kg ha^-1^ *fb* tank mix application of pyrithiobac-sodium 62.5 g/ha +quiza- lofop ethyl 50 g/ha as PoE (2-3 weed leaf stage) *fb* directed spray (inter-row) of paraquat 24% SL 0.5 kg/ha at 55 DAS. | Diuron PE 0.75  kg/ha *fb* mechanical brush cutter twice at 25  and 60 DAS. | One hand weeding was done after the critical period of crop-weed competit-ion *i.e.* between 45-50 days after sowing). | Atrazine 1.0 kg/ha + paraquat 600 g/ha PE *fb* tembotrione 120 g/ha at  20-25 DAS  as PoE (T_2_, T_3_). Atrazine 1 kg ha^-1^ PE *fb* tembotrione 120g/ha at 20-25 DAS as PoE (T_1_). | Atrazine 1.0 kg/ha  + paraquat 600 g/ha PE *fb* tembotrione 120 g/ha at 20-25 DAS as PoE (T_2_, T_3_). Atrazine 1.0 kg/ha PE *fb* tembotrione 120g/ha at 20-25 DAS at PoE (T_1)_.  **rotated with**  Atrazine 1.0 kg/ha  + paraquat 600 g/ha PE *fb* halosulfuron- methyl 67.5 g/ha at 20-25 DAS as PoE (T_2_, T_3_). Atrazine 1.0 kg/ha PE *fb* halo-sulfuron methyl 67.5 g/ha at 20-25 DAS as PoE (T_1_). | Tembotrione 120 g/ha & Atrazine 50% WP 0.5 kg/ha both applied as early post-emergence) EPoE *fb* brush cutter at 40 DAS. | One hand weeding was done after the critical period of crop-weed competit-ion *i.e.* between 45-50 days after sowing). |
| **T_2_** |  |  |  |  |  |  |  |  |
| **T_3_** |  |  |  |  |  |  |  |  |

T_1_ = conventional tillage (cotton) – conventional tillage (maize) – Fallow (No *Sesbania rostrata*), T_2_ = conventional tillage (cotton) – zero tillage (maize) – zero tillage (*Sesbania rostrata*), T_3_= zero tillage (cotton) + *Sesbania rostrata* residues (*Sr*R) – zero tillage (maize) + cotton residues (CR) – zero tillage (*Sesbania rostrata*) + maize stubbles (MS), IWM= integrated weed management.

**Table supplementary 2:** Experiment particulars

| Gross plot area | 9.6 m × 5.4 m |
| --- | --- |
| Net plot area | 8.4 m × 3.6 m |
| Spacing for cotton | 90 cm × 60 cm |
| Spacing for maize | 60 cm × 25 cm |
| Spacing for *Sesbania* | 30 cm spaced solid rows |

**Table supplementary 3:** Characteristics of cotton, maize and *Sesbania* cultivars used

| **S. No** | **Characters** | **Cotton** | **Maize** | ***Sesbania*** |
| --- | --- | --- | --- | --- |
| 1 | Variety | *Sadanand* | DHM 117 | – |
| 2 | Duration (days) | 180 days | Medium,110-120 days | 40 – 60 days |
| 3 | Season | Monsoon | Winter | Summer |
| 4 | Habit | Tall, erect and open | Sturdy, tall and non-  lodging | Tall and branched |
| 5 | Grain type | Ovate and large sized balls | Flint seeds in cob | Linear or slightly curved pods |
| 6 | Average yield | 2400 – 2800  (kg ha^-1^) | 7500  (kg ha^-1^) | 10.5  (tons biomass ha^-1^) |

**Table supplementary 4:** The dates for sowing and harvesting of cotton-maize- *Sesbania rostrata* from

2020 to 2023 in conservation agriculture

| Date of sowing cotton | 15/07/2020, 31/07/2021 and 07/07/2022 |
| --- | --- |
| Date of sowing maize | 05/01/2021, 07/01/2022 and 06/01/2023 |
| Date of sowing *Sesbania* | 18/05/2021, 03/06/2022 and 20/05/2023 |
| Date of final harvesting of cotton | 22/12/2020, 24/12/2021 and 23/12/2022 |
| Date of harvesting maize | 14/05/2021, 28/05/2022 and 08/05/2023 |
| Date of harvesting *Sesbania* | 17/06/2021, 02/07/2022 and 19/06/2023  by knocking it down and removing  in T_2_ and shredded for retention in T_3_ as surface mulch. |

**Table supplementary 5:** Kernel/ grain yield of maize and seed cotton yield as influenced by tillage

practices and weed management (WM) options in conservation agriculture after 3^rd^ year (8^th^ crop cycle).

| **Treatment Interaction** | **WM** | **kernel yield**  **(kg ha-₁)** | **Seed cotton yield**  **(kg ha-₁)** | |
| --- | --- | --- | --- | --- |
| **Tillage** |  |  |  |  |
| T_1_: CT(C)-CT(M)-Fallow (N*Sr*) | W_1_ | 6822 | 1657 | |
|  | W_2_ | 6854 | 1693 | |
|  | W_3_ | 6354 | 1954 | |
|  | W_4_ | 4025 | 610 | |
|  | W_1_ | 7133 | 1811 | |
| T_2_: CT(C)-ZT(M)-ZT(*Sr*) | W_2_ | 7662 | 1830 | |
|  | W_3_ | 6558 | 2091 | |
|  | W_4_ | 3559 | 672 | |
| T_3_: ZT(C)+*Sr*R-ZT(M)+CR-ZT(*Sr*)+MS | W_1_ | 7780 | 1899 | |
|  | W_2_ | 7456 | 1912 | |
|  | W_3_ | 7253 | 2223 | |
|  | W_4_ | 4713 | 697 | |
| **Tillage practices** | | | | |
| T_1_: CT(C)-CT(M)-Fallow (N*Sr*) | 6014 | | 1478 | |
| T_2_: CT(C)-ZT(M)-ZT(*Sr*) | 6228 | | 1601 | |
| T_3_: ZT(C)+*Sr*R-ZT(M)+CR-ZT(*Sr*)+MS | 6801 | | 1683 | |
| **Weed Management options** | | | | |
| W_1_- Chemical weed control | 7245 | | 1789 | |
| W_2_- Herbicide rotation | 7324 | | 1812 | |
| W_3_- IWM | 6722 | | 2089 | |
| W_4_- Single hand-weeded control | 4099 | | 660 | |
|  | **SE(m)±** | **CD(P=0.05)** | **SE(m)±** | **CD(P=0.05)** |
| **Tillage** | 144.83 | 568.66 | 38.56 | 151.43 |
| **Weed Management** | 126.98 | 377.28 | 32.22 | 95.75 |
| **Interactions** | | | | |
| **W at same level of T** | 219.94 | NS | 55.82 | NS |
| **T at same level of W** | 239.28 | NS | 61.84 | NS |

T_1_ = conventional tillage (cotton) – conventional tillage (maize) – Fallow (No *Sesbania rostrata*), T_2_ = conventional tillage (cotton) – zero tillage (maize) – zero tillage (*Sesbania rostrata*), T_3_= zero tillage (cotton)+*Sesbania rostrata* residues (*Sr*R)– zero tillage (maize) + cotton residues (CR) – zero tillage (*Sesbania rostrata*) + maize stubbles (MS), T= TillIage, W=Weed management, IWM=Integrated weed management, CD (P= 0.05) = critical difference at 5% probability level, NS = non-significant, SE(m) = standard error of the mean.

**Supplementary Figure 1:** Weekly-base mean meteorological observations during maize development.


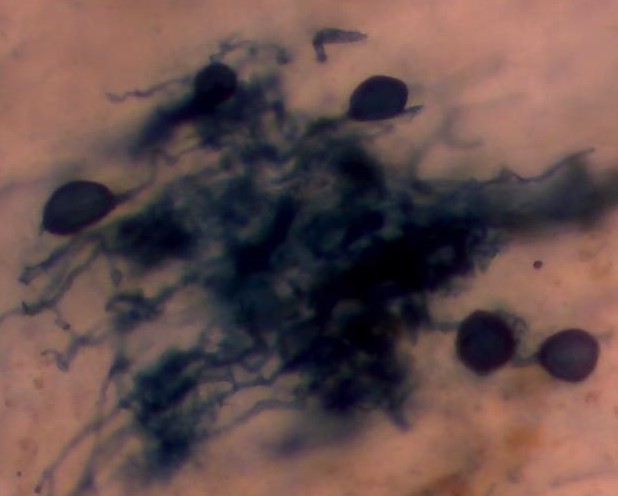

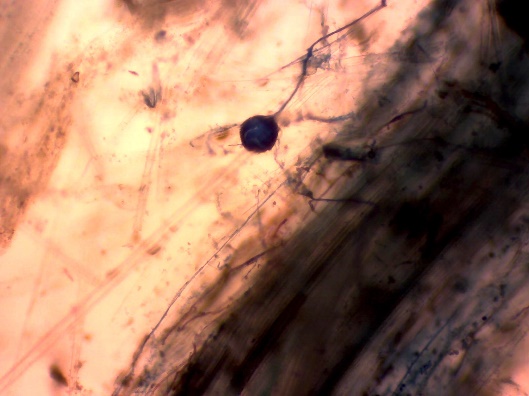


**Mg. 10X**

**Mg. 20X**

**a**

**b**

**c**

**a**

**b**

**c**


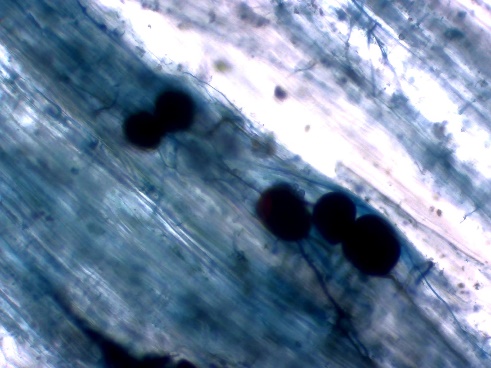

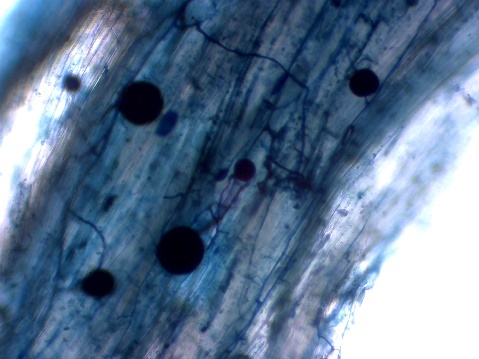


**Mg. 10X**

**Mg. 10X**

**a**

**b**

**c**

**a**

**b**

**c**


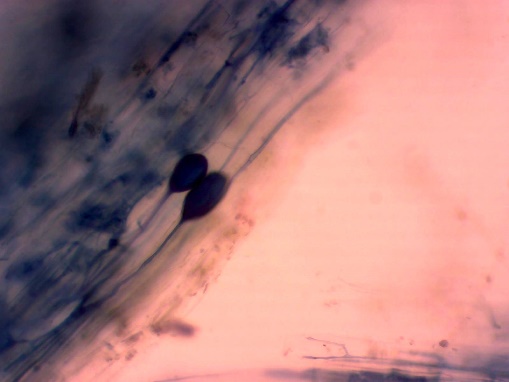


**Mg. 20X**

**a**

**b**

**c**

**Supplementary Figure 2:** Overview of AMF across treatment combinations at 60 days after sowing of maize crop. **Mg.** = magnification; **a**= Vesicle; **b**= Hyphae; **c**= arbuscules
